# Of mice and men: Laboratory murine models for recapitulating the immunosuppression of human sepsis

**DOI:** 10.3389/fimmu.2022.956448

**Published:** 2022-08-05

**Authors:** Ning Wang, Yongling Lu, Jiang Zheng, Xin Liu

**Affiliations:** ^1^ West China Biopharm Research Institute, West China Hospital, Sichuan University, Chengdu, China; ^2^ Medical Research Center, Southwest Hospital, Army Military Medical University, Chongqing, China

**Keywords:** sepsis, immunosuppression, murine models, immunopathy, preclinical study, LPS tolerance

## Abstract

Prolonged immunosuppression is increasingly recognized as the major cause of late phase and long-term mortality in sepsis. Numerous murine models with different paradigms, such as lipopolysaccharide injection, bacterial inoculation, and barrier disruption, have been used to explore the pathogenesis of immunosuppression in sepsis or to test the efficacy of potential therapeutic agents. Nonetheless, the reproducibility and translational value of such models are often questioned, owing to a highly heterogeneric, complex, and dynamic nature of immunopathology in human sepsis, which cannot be consistently and stably recapitulated in mice. Despite of the inherent discrepancies that exist between mice and humans, we can increase the feasibility of murine models by minimizing inconsistency and increasing their clinical relevance. In this mini review, we summarize the current knowledge of murine models that are most commonly used to investigate sepsis-induced immunopathology, highlighting their strengths and limitations in mimicking the dysregulated immune response encountered in human sepsis. We also propose potential directions for refining murine sepsis models, such as reducing experimental inconsistencies, increasing the clinical relevance, and enhancing immunological similarities between mice and humans; such modifications may optimize the value of murine models in meeting research and translational demands when applied in studies of sepsis-induced immunosuppression.

## Introduction

Sepsis is defined as a life-threatening organ dysfunction caused by a dysregulated host response to infection (1). As a leading cause of death in intensive care units (ICUs), sepsis affected an estimated 48.9 million people in 2017, with a death toll surpassing 10 million (2). Due to advances in supportive care, the in-hospital mortality during the early stages of sepsis has been significantly reduced, resulting in a dramatic increase in late-phase sepsis patients and sepsis survivors (3). Persistent immunosuppression is a hallmark of late-phase sepsis and post-sepsis syndrome, which disrupts the host’s antimicrobial response against secondary infection, culminating in organ dysfunction and death (4). Notably, patients are increasingly admitted to hospital with concomitant diseases or immune compromised conditions, which increases their risk of developing sepsis (5) or is associated with poor prognosis post-sepsis (6, 7). Consequently, extensive studies have been performed to uncover the mechanisms that drive immunosuppression in sepsis (8). Moreover, immunostimulatory strategies that aim to reverse the immunocompromised status for patients suffering sepsis, are also being increasingly appraised in experimental animals or in human subjects (9).

Many insights into the pathogenesis of sepsis, including the development of immunosuppression, were first derived from animal models (7, 10, 11). The pharmacological evaluation of immunostimulatory agents has always been initiated in preclinical studies (9). However, the reproducibility and translational feasibility of animal studies are often questioned due to inherent immunological discrepancies between animals and humans (11). Inconsistencies in modeling procedures may further broaden the gaps between modeled and clinical sepsis. Despite of their inevitable deficiencies, the validity and clinical relevance of preclinical sepsis models can be improved by standardizing the modeling procedures and refining the modeling strategies. In this mini review, we describe the major types of animal models used to mimic the immunosuppression observed in human sepsis, highlighting their strengths and limitations. We also propose potential directions for improving the quality and value of preclinical models of sepsis. Given that most studies of sepsis are performed in mice (due to numerous advantages such as ease of accessibility and handling, convenience of examining the immune response, and feasibility of genetical manipulation) (11), this mini review exclusively discusses the use of laboratory murine models used to specifically recapitulate the immunosuppression observed in human sepsis.

## Immunopathogenesis in sepsis

Compelling experimental and clinical evidence has indicated that elements of both pro-inflammatory and anti-inflammatory responses occur early and simultaneously in sepsis (12, 13). Typically, a rapid onset of pro-inflammatory reactions, including the excessive release of pro-inflammatory cytokines and the hyperactivation of the complement system, the coagulation system and the endothelial system, are provoked by the activation of PAMPs and DAMPs, giving rise to life-threatening organ injuries at the early stage of sepsis (14, 15). Meanwhile, an adaptive anti-inflammatory phenotype is also upregulated which alters the status of innate immune cells and induce apoptosis and anergy in lymphocytes, leading to a long-term immunosuppression that characterize late sepsis or post-sepsis sequelae (15). Of note, the signs of immunoparalysis are much early or even appear first after sepsis in immunocompromised patients (15). Given that the majority of patients are likely to survive the early stage of sepsis while an expansion of aging people or other population predisposing to an immunosuppressive status tend to be more prone to sepsis, increasing awareness has been given to understanding the mechanisms that drive sepsis-induced immunosuppression (7). A number of key events, such as transcriptional reprogramming, epigenetic modifications and metabolic disorders, have been demonstrated to promote leukocyte tolerance (reduced cytokine production and impaired antigen presentation), increase the expression of inhibitory checkpoint molecules (e.g., programmed cell death protein 1 (PD-1), programmed cell death ligand 1 (PD-L1), and cytotoxic T - lymphocyte antigen 4 CTLA-1) or suppressive immune cells (e.g., regulatory T cells (Tregs) and myeloid-derived suppressor cells (MDSCs)) and induce anergy or death in lymphocytes (8, 14). Meanwhile, the inevitable use of immunosuppressive agents, such as norepinephrine and hydrocortisone, may further deteriorate the immunocompromised status in sepsis (12). Consequently, a broad and persistent dysfunction occurs in host immune responses, leading to increased susceptibility for low-virulent bacterial, fungal and viral pathogens (15). This results in unresolved septic foci, incapability to combat secondary or nosocomial infections and other multiple complications that cause multiple organ dysfunction syndromes (MODS), extend hospital length of stay, and may even leads to death in the late phase or after discharge (12).

## Murine models use to recapitulate immunosuppression in sepsis

**Figure 1:**
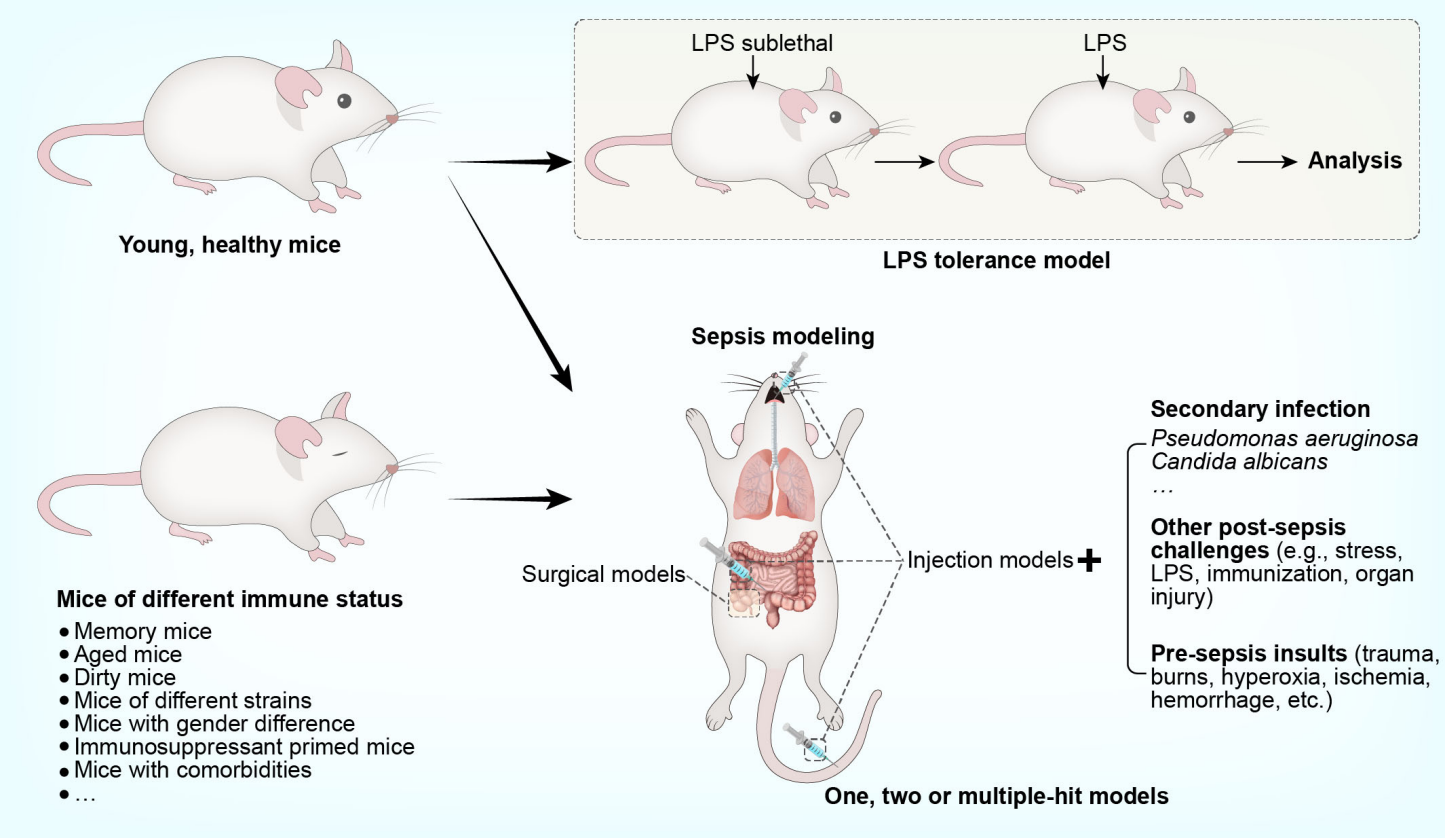
Murine models that recapitulate immunosuppression in human sepsis.

Sepsis is characterized by a profound shift from an overwhelmingly hyperinflammatory state towards a broad defect in both innate and adaptive immunity (8). Therefore, experimental studies are performed in murine models, allowing a natural course of sepsis-induced immunosuppression (16). Given the susceptibility of immunocompromised patients to secondary infection, microbial insults are often additionally imposed on septic animals; the resulting models are termed ‘two-hit’ or ‘double-hit’ models (17). In some cases, a pre-sepsis insult (e.g., trauma, burns, hyperoxia, ischemia, or hemorrhage) or other post-sepsis challenges (e.g., stress, lipopolysaccharide (LPS) exposure, immunization, or organ injury) may also be involved to create a model with more than ‘two-hit’. To understand specific features of sepsis-associated immunosuppression, murine models with special features are sometimes developed. A good example is the use of the LPS tolerance model to reveal alterations reminiscent of leukocyte reprogramming in human sepsis (8). Further, compelling evidence has suggested an obvious immunological gap in the pre-sepsis stage between animal models and human sepsis (7). Therefore, sepsis is modeled in mice under different immunological conditions, such as memory mice, dirty mice, aged mice, and mice with genetic or gender-specific differences. Considering that human sepsis is often associated with the use of immunosuppressants or the presence of concomitant diseases, sepsis is also modeled in mice primed with immunosuppressant or bearing comorbidities. An overview of the models used to specifically recapitulate sepsis-induced immunosuppression, is provided in **Figure 1**. The key steps involved in establishing these models, along with their main advantages and disadvantages, are addressed in detail below and summarized in **Tables 1** and **2**.

**Table 1:** Comparison of major modeling methodologies used to generate murine models of sepsis-associated immunosuppression.

Model type	Modeling methods	Clinical relevant manifestations	Features of immunosuppression	Model strengths	Model weaknesses
**The one-hit model**	• Intranasal or intraperitoneal pathogen inoculum • Intraperitoneal feces injection • CLP or CASP	• Clinically relevant to late sepsis from abdominal or lung infection • Sublethal or low-mortality • Low-grade or persistent cytokine production, moderate hypotension and organ injury, splenic myelopoiesis and prolonged immunosuppression (PICS)	• Immune anergy • Lymphopenia • Elevated inhibitory markers (e.g., PD-L1) • Elevated suppressive immune cells (e.g., Treg, MDSCs, etc.)	• Requiring no other modeling methods • Mimicking a natural development of immunosuppression in sepsis	• Diversified inflammatory and immune profiles after modeling • Without secondary insult to reflect immunosuppression
**The two-hit model**	• First hit: sublethal septic insult (CLP most commonly) • Second hit: Bacterial (e.g., *P. aeruginosa, S. aureus*) or fungal (e.g. *C. albicans*) infection or other post-sepsis challenges (e.g., stress, LPS, immunization, organ injury)	• Clinically relevant to sepsis with secondary infection • First hit: Similar mortality, cytokines, and organ injury to one-hit models • Second hit: Increased pathogen load, reduced cytokine production, worsened organ injury, and elevated mortality	• Similar to one-hit models • Susceptible to secondary infection	• Recapitulating immunosuppression in mice surviving early sepsis • Similar to secondary infection or other immune deficiencies in human sepsis	• Different status after the first hit • Lack of standardized second hit method (e.g., microbial species, time course, and dosage)
**The more than two-hit model**	• Two-hit models with pre-sepsis insult (trauma, burns, hyperoxia, ischemia, hemorrhage, etc.)	• Clinically relevant to sepsis secondary to multiple injuries • Stronger inflammation, organ injury than one-hit or two-hit models	• Worse immunosuppression than one-hit or two-hit models	• Mimicking immunosuppression by both combined injury and sepsis	• Higher modeling inconsistency • Heterogeneous immune responses created by different insults
**The LPS tolerance**	• Priming: repeated exposure to sublethal LPS • Re-challenge: lethal dosage of LPS	• Clinically relevant to endotoxemia • Reprogrammed cytokines (proinflammatory ↓, anti-inflammatory ↑) • Organ mildly injured in priming while protected in re-challenge • Susceptible to secondary infection	• Monocyte exhaustion (phagocytosis↑, antigen presentation↓, bacterial killing↓) • Elevated inhibitory markers and suppressive cells	• Recapitulating leukocyte reprogramming in human sepsis	• Focusing on monocytes and macrophages only • Different from immunosuppression in human sepsis (IL-10↓, phagocytosis↓)

CLP, cecal ligation and puncture; CASP, colon ascendens stent peritonitis; PICS, persistent inflammation, immunosuppression and catabolism syndrome; Tregs, regulatory T-cells; MDSCs, myeloid-derived suppressor cells.

**Table 2:** Summary of mouse models with specific immunologically characteristics to study immunosuppression in sepsis.

Model type	Background	Immune status	Manifestations in sepsis modeling
**Memory mice**	Pathogenic or antigenic pre-exposure to develop immune memory	Trained immunity Enhanced antigen-specific memory T-cells Reprogramming of myeloid cells	Clinically relevant to reinfection or vaccination Augmented inflammatory response Enhanced protection against secondary infection
**Aged mice**	>18 months old	Immune cell senescence Chronic inflammation, persistent immunosuppression	Clinically relevant to sepsis in the elderly Insufficient myeloid response, T-cell exhaustion Heavier organ dysfunction and reduced survival
**Dirty mice**	Exposure to microbes by co-housing, sequential infection, microbiota transfer, and rewilding	Experienced immunity (memory, differentiation in T-cells) Natural microbiota and pathogens	Better recapitulation of human immunity in sepsis Enhanced inflammation and protection against infection
**Mice from different strains**	Genetic variation (Th1 vs. Th2) Genetic heterogeneity (inbred vs. outbred)	Higher immunosuppression in Th2 (BALB/c, etc.) than in Th1 (C57BL/6, etc.) strains due to genetic variation Lower immunosuppression in outbred (CD-1, etc.) than in inbred (C57BL/6J, etc.) strains due to genetic heterogeneity	More unresolved inflammation, impaired bacterial clearance and susceptibility to infection in Th2 strains than in Th2 strains Lower Th1 cytokines and more susceptible to infection in inbred than in outbred strains
**Mice with gender difference**	Male vs. female	Depressed cellular immune responses in males while unchanged or enhanced in females under stress conditions Immunosuppressive male sex hormones vs. immunostimulatory female sex hormones	Clinically relevant to gender-associated variations in sepsis Higher inflammation and sustained immune response, enhanced bacterial killing and increased survival in female than male mice
**Immunosuppressant- primed mice**	Preconditioning with immunosuppressants (e.g., glucocorticoids, calcineurin inhibitors, and fingolimod)	Endotoxin tolerance (glucocorticoids) Lymphopenia and T-cell dysfunction (calcineurin inhibitors, fingolimod)	Clinically relevant to predisposed immunosuppression Decreased inflammatory cytokine release Heavier organ damage and higher bacterial load
**Mice with comorbidities**	Preconditioned illness (autoimmunity disease, obesity, cancer, and NAFLD) before sepsis modeling	Pronounced T-cell apoptosis and Treg expansion	Clinically relevant to sepsis with comorbidities Elevated morbidity and mortality Increased gut permeability, persistent inflammation, aggravated organ injury, and more prone to immunosuppression

### The ‘one-hit’ model

The one-hit model is an exactly routine sepsis model, which can be simply categorized into injection models and surgical models by the way sepsis is recapitulated. The injection models are established by giving an exogenous toxin (e.g. lipopolysaccharide (LPS), a viable pathogen (e.g. *Escherichia coli*), and feces or other pathogen containing materials (18). The surgical models are made by disrupting the endogenous barriers *via* surgery, which thereby induces local infection and sepsis. Cecal ligation and puncture (CLP) model and colon ascendens stent peritonitis (CASP) model are typical surgical models that reproduce abdominal sepsis *via* intra-abdominal surgery (18). Despite similar lethality in these models, intravenous injection of lethal doses of toxins or live pathogens induces a rapid and severe systemic proinflammatory response rather than a low-grade inflammation, accompanied with persistent immunosuppression (19). In contrast, local infection models established by injection, implantation or surgery demonstrate attenuated inflammatory response and increased tendency to develop immunosuppression (20, 21). Typically, the CLP model is most widely used to demonstrate ongoing immune suppression, including splenocyte apoptosis (22), lymphopenia (23) and expansion of Tregs (24) and MDSCs (25). Immunosuppression can be directly evaluated by using one-hit models, allowing a natural sepsis course without other modeling methods. However, a diversified inflammatory and immune profiles may exist with different modeling strategies. Moreover, the lack of a secondary insult makes them insufficient to reflect host response in an immunosuppressive state.

### The ‘two-hit’ model

To induce immunosuppression following sepsis, model mice are first made to develop sepsis (the first hit) and then challenged with a secondary infection (the second hit). Indeed, two-hit mice are demonstrated to exhibit increased bacterial load and lower inflammatory resolution, thereby recapitulating the nosocomial infection observed at the prolonged immunosuppressive stage post-sepsis (26, 27). The first hit is typically performed using routine sepsis modeling methods, either by extrinsic pathogen inoculation or surgical approaches that establish intrinsic infection. During the second hit procedure, a clinically relevant pathogen is commonly injected into mice to mimic the secondary infection encountered in human sepsis.

Specifically, sublethal or low-lethal intraperitoneal surgery in the form of cecal ligation and puncture (CLP) is most widely applied to perform the first hit. Then a secondary pneumonia or systemic infection is established *via* the administration of *Pseudomonas aeruginosa*, *Candida albicans* or other opportunistic pathogens that are non-lethal in healthy or sham mice but induce high mortality in the model mice (26, 28). Generally, the first-hit by CLP creates a similarly immunosuppressive status in mice, which consistently renders them more susceptible to secondary infection by either *Pseudomonas aeruginosa*, *Staphylococcus aureus* or *Candida albicans*. However, bacterial infection is preferentially given *via* the intranasal route which is clinically relevant of hospital-acquired pneumonia secondary to abdominal sepsis or injuries. In contrast, fungal pathogens are commonly injected intravenously to mimic disseminated infection in human sepsis (29). In addition to secondary infection, post-CLP mice may suffer LPS injection or daily chronic stress to induce an aggravated inflammation response that recapitulates persistent inflammation, immunosuppression, and catabolism syndrome (PICS) after sepsis (30, 31). Other secondary insult, such as antigen immunization is also given after CLP to evaluate the adaptive immunosuppression, including Treg expansion and reduced memory CD4+ T cells (32, 33). Moreover, CLP mice are subjected to second challenge of organ injury or wound, which reflects either the impaired organ protection or wound healing due to immunosuppressive state (34, 35). Given that pneumonia is a leading cause of sepsis, the first hit is sometimes performed by intratracheal bacterial inoculation to replicate a natural immunodeficient state that develops in pneumonia-induced sepsis. A second hit of bacterial or viral infection is then administered *via* inoculated to examine the paralyzed immune state characteristic of the post-sepsis period (36).

Two-hit models mimic the natural course of immunosuppression development in sepsis, and therefore, they best characterize the systemic immunopathology of human sepsis. However, two-hit mouse models can be generated under varying conditions and the first hit can be created using different methods. Additionally, the microbial species used, as well as the time course and dosage of second hit have not been standardized, which may further increase the inconsistency of outcomes and hinder comparisons between different laboratories. In some experiments, two-hit models are coupled with pre-sepsis insult, such as trauma, burns, hyperoxia, ischemia and hemorrhage (37–39). These models may refer as ‘more than two-hit’ models, with stronger inflammation and organ injury are demonstrated in these models compared with one-hit or two-hit models. These models are clinically relevant to sepsis secondary to multiple injuries. However, they may display higher modeling inconsistency due to more heterogeneous immune responses by different pre-sepsis insults.

### The LPS tolerance model

The injection of LPS is extensively used to induce sepsis in experimental models. However, studies using lethal dosages of LPS are no longer convincing due to the rapid kinetics, extreme inflammation, and immediate cardiovascular collapse, which are dramatically different from human sepsis that originates from localized infection (18). Nevertheless, the LPS tolerance model, which is induced by repeated exposure with sublethal doses of LPS, resembles a key feature of innate immune system paralysis in human sepsis, known as leukocyte reprogramming (40). Leukocyte reprogramming defines an adaptive immune response that is associated with a decline in proinflammatory cytokine production and the downregulation of surface HLA-DR expression on leukocytes upon stimulation with microbial agonists like LPS; this phenomenon is also known as LPS tolerance. Leukocyte reprogramming can also be detected in LPS-tolerant mice (14). Therefore, this model is valuable for exploring the mechanisms of long-term adaptation of innate immune cells following excessive inflammation to immunosuppression in sepsis, such as epigenetic reprogramming, autophagy impairment, decrease in the levels of activating cell surface molecules and the upregulation of negative regulatory factors (40, 41). However, some studies suggest that the phagocytic function and pathogen killing capacity of monocytes/macrophages in this model are enhanced while the anti-inflammatory mediators such as interleukin (IL)-10 are upregulated (8). These phenotypes differ from clinical observations of immunosuppression in human sepsis, which is overall aberrant in cytokine production and is associated with a refractory response to secondary infection (4). Furthermore, the immune state observed in the murine LPS tolerance model arises specifically in response to LPS stimulation rather than recapitulating the immunosuppression observed in human sepsis, which is a consequence of polymicrobial infection.

### The use of mice with specific immunological features to model the immunosuppression observed in human sepsis

Unlike young, healthy, naïve mice that are often used to recapitulate human sepsis, patients with sepsis have heterogeneous characteristics such as discrepant age, gender, living environment, genetic background, and immunological status. Therefore, attempts have been made to increase the clinical relevance of murine models by modifying their immunological profiles or considering different immunological backgrounds. For example, standard laboratory mice are hygienic and lack effector-differentiated and mucosal memory T-cells, due to being housed under specific pathogen-free conditions (42). Therefore, memory mice are made to develop immune memory by pre-exposure to specific pathogenic or antigenic stimuli. Memory mice are also clinically relevant models for the study of reinfection or vaccination. They display raised inflammatory responses and a higher level of protection against secondary infection following sepsis induction. Likewise, dirty mouse models are also introduced by exposure to microbes *via* co-housing, sequential infection, the transfer of microbiota, and rewilding. Dirty mice have an experienced immune system and are exposed to naturally-occurring microbiota and pathogens, meaning that their inflammatory response and protection against infection are more relevant to human immunity during sepsis. Interspecies immune discrepancies are ascribed to genetic variations of laboratory mice that profoundly affect the responsiveness of immunity in sepsis., mice with genetic variations (Th1 vs. Th2) or genetic heterogeneity (Inbred vs. outbred) differ in their immune response to sepsis. Typically, higher levels of immunosuppression can be detected in Th2 mice (e.g., BALB/c) than in Th1 (e.g., C57BL/6) strains due to genetic variations (43). In addition, lower-level immunosuppression is found in outbred (e.g., CD-1) than in inbred (e.g., C57BL/6J) strains, due to genetic heterogeneity (44). Therefore, it is necessary to consider these variations when recapitulating sepsis in murine models.

Animals uses as models are typically normal in their immunological status prior sepsis modeling. However, patients are more likely to suffer from the effects of sepsis when they are either already immunodeficient or develop early immunosuppression post-infection. The immunological susceptibility to sepsis has prompted attempts to establish pre-sepsis immunosuppression in model animals *via* the administration of immunosuppressants. Indeed, immunosuppressants such as cyclophosphamide or cyclosporine were given to mice to induce a pre-existing immunosuppressive state. When pathogens were injected into these mice, the animals developed sepsis in similar fashion to immunocompromised patients (45, 46). However, pre-existing immunosuppressive status may not accurately replicate the natural course and severity of sepsis-induced immunoparalysis. The release of proinflammatory mediators, for instance, remains persistently low in this type of model, and the transition from hyperinflammation towards immunosuppression, as seen in human sepsis, is not observed (45). Moreover, cyclosporine mainly impairs T lymphocyte activity, rather than suppress the overall immune response, which also differs from the immunopathy encountered in sepsis (45).

Sepsis is often associated with co-morbidities (e.g., trauma, obesity, and cancer), causing patients to become more predisposed to immunosuppression in sepsis (47, 48). Therefore, researchers have modeled sepsis in animals bearing these co-morbidities. For instance, traumatic hemorrhage was induced in mice prior to CLP to examine its impact on the immune status and the ability to survive the subsequent onset of sepsis (49). Other studies have modelled sepsis-associated immunosuppression in obese mice (50) or tumor-bearing mice (51, 52), which were designed to evaluate the impact of co-morbidities on the development of sepsis and sepsis-associated immunoparalysis. Such studies are important for broadening our understanding of the heterogeneity of sepsis but are not ideal for delineating the mechanisms of immunosuppression, which can be attributed to sepsis or the comorbidity. In addition, animals with pre-existing diseases often produce more heterogenous experimental outcomes, thereby demanding more standardized and elaborate modeling procedures.

## Strengths and limitations of modeling sepsis-induced immunosuppression in mice

### How do murine models accurately replicate the immunological alterations in human sepsis?

Mice and humans share a highly homologous genetic background (>80% conserved synteny), meaning that the discovery of murine genes and corresponding phenotypes often applicable to humans (53–55). Shay et al. (56) compared the genome-wide transcriptional compendia of humans and mice, and founds that the resting and activated immune cells of both organisms shared a conserved transcriptional program and associated regulatory mechanisms. An earlier collaborative research program termed Glue Grant, which addressed how murine models mirror human disease in sepsis and trauma (57), described more specifically the similarities in both species upon endotoxin challenge, including the appearance of lymphopenia, which characterizes immunosuppression in sepsis (58). A series of subsequent studies further compared interspecies data derived from Glue Grant or other databases (e.g., the Immunological Genome Project). Although the overall transcriptomic association between mouse injury models and human inflammatory diseases (such as trauma, burns and sepsis) was weak (59, 60), the signaling pathways involved in early inflammation and innate/adaptive immunity were similar in both species (60). In addition, a number of pathways/biogroups that reflect both excessive inflammation and immunosuppression, such as enhanced cytokine signaling and suppressed lymphocyte differentiation, are altered in both mice and humans consistently (61). A more recent study further demonstrated that the gene expression patterns in LPS-stimulated mouse peritoneal cells, including genes associated with immunosuppression, are similar to genes upregulated in human cells stimulated with LPS *in vitro* or cells isolated from septic patients (62). These findings suggest the feasibility of murine models to accurately replicate the immunological alterations in human sepsis.

Specific murine models reflect the features of immunosuppression in human sepsis, either in part or in whole. For example, a mixture of both pro- and anti-inflammatory reactions, accompanied with subsequent dysfunction of adaptive immune responses that characterize the immunosuppression of human sepsis, has been demonstrated in murine CLP models (28, 63). Moreover, the phenomenon of leukocyte reprogramming in human sepsis, as indicated by diminished cytokine release and impaired antigen presentation, can also be similarly detected in murine LPS tolerance model (40). Other similarities due to the influence by different immunological backgrounds, such as age and gender, have also been identified in both mice and humans. For example, the increased production of immunosuppressive cytokines and the impaired CD4+ T cell proliferation were similarly detected in elderly sepsis patients and CLP mice (64). Furthermore, clinical and experimental studies have both indicated that female mice and patients are immunologically more competent than male subjects upon sepsis insult, which renders the transition towards immunosuppression and decreases the susceptibility to secondary infection (65).

### How do murine models advance our understanding of immunosuppression in sepsis?

First, the principal immunological players involved in sepsis were predominantly discovered in mice and only later identified in humans (55). For instance, toll-like receptor 4 (TLR4) was initially characterized in mice as the pattern recognition receptor (PRR) for LPS, and represents a significant milestone in improving our understanding of the immunopathology of sepsis (66). Likewise, the discovery of the metabolic and epigenetic rewiring mechanism that drives the shift towards immunosuppression in sepsis was also initially made using mouse models (67, 68). Other major immunological breakthroughs in sepsis, such as the characterization of immunosuppressive cells (e.g., regulatory T-cells, Tregs; and myeloid-derived suppressor cells, MDSCs) and immune checkpoints (e.g., programmed cell death protein-1, PD-1; and cytotoxic T-lymphocyte antigen 4, CTLA-4), occurred initially in models, before further verification of their importance in human sepsis (69–71). Second, our knowledge of sepsis immunology in humans is almost exclusively derived from studies using blood samples. However, increasing importance has been attributed to the roles of tissue and organ immunology in sepsis (7). In this regard, it may be more valuable to examine immunological profiles at the site of infection rather than solely in the blood (72). A variety of sample types can be extracted from murine models, thereby facilitating the characterization of compartment-specific immunopathy in sepsis. For instance, the functionality of alveolar macrophages and microglial cells are primed or unaltered, respectively, in the LPS injection or CLP models, compared to the tolerance induction observed in peritoneal cells and splenocytes (7). In addition, Komegae et al. (73) recently reported the site-specific responsiveness of alveolar, peritoneal, and adipose-associated macrophages to bacterial LPS, as indicated by the different tumor necrosis factor alpha (TNF-α)/IL-10 ratios that are indicative of the immunosuppressive status. Such results cannot be obtained *via* the study of peripheral blood samples alone.

Third, murine models are extensively used preclinical studies to inform future clinical trials or support the implementation guidelines for the management of sepsis survivors (74). In particular, attempts have been made in animal models to reverse the immunocompromised status or boost immunity with immunostimulants by means of cytokines, peptides, small molecule compounds, and cell transfer strategies (14). For example, pharmacological or genetic approaches to suppressing lymphocyte apoptosis have been verified in experimental models, aiming to evaluate the potential of reducing sepsis lethality by targeting apoptosis in lymphocytes (14).

### How to understand the limitation of using murine models in studying immunosuppression in sepsis?

Mouse models have improved our understanding of the immune profiles that exist in sepsis. However, there are a number of discrepancies that interfere with the recapitulation of the immunosuppression observed in human sepsis. Of note, these discrepancies may either result from their inherently-distinct immune responses or otherwise are induced by inappropriate experimental procedures during sepsis modeling.

First, the precise immunological changes are not consistent between murine models and human sepsis (75). In fact, the dynamics of the immunological response is much more rapid and intense in murine sepsis models, and lead to a short course of disease. However, human sepsis is often chronic and immunological alterations, including the onset of immunosuppression, is persistent or repeated. Further, murine models that recapitulate sepsis induced by a certain kind of insult or at a specific infection site can only model a subtype of sepsis or demonstrate one aspect of the pathophysiological changes that are encountered in human sepsis. To illustrate, induction of LPS tolerance may only mimic compartmentalized immunological changes in sepsis. However, a modeling strategy that induces sepsis by targeting different tissue sites may significantly affect the immune response in sepsis (20). Similarly, the administration of IL-10, an immunosuppressive cytokine, has been shown to protect model mice from abdominal sepsis but led to the deterioration of mice with pulmonary sepsis (20). In this regard, when investigating the pathology or treatment of immunosuppression in sepsis, results from different models should be interpreted with caution.

**Figure 2:**
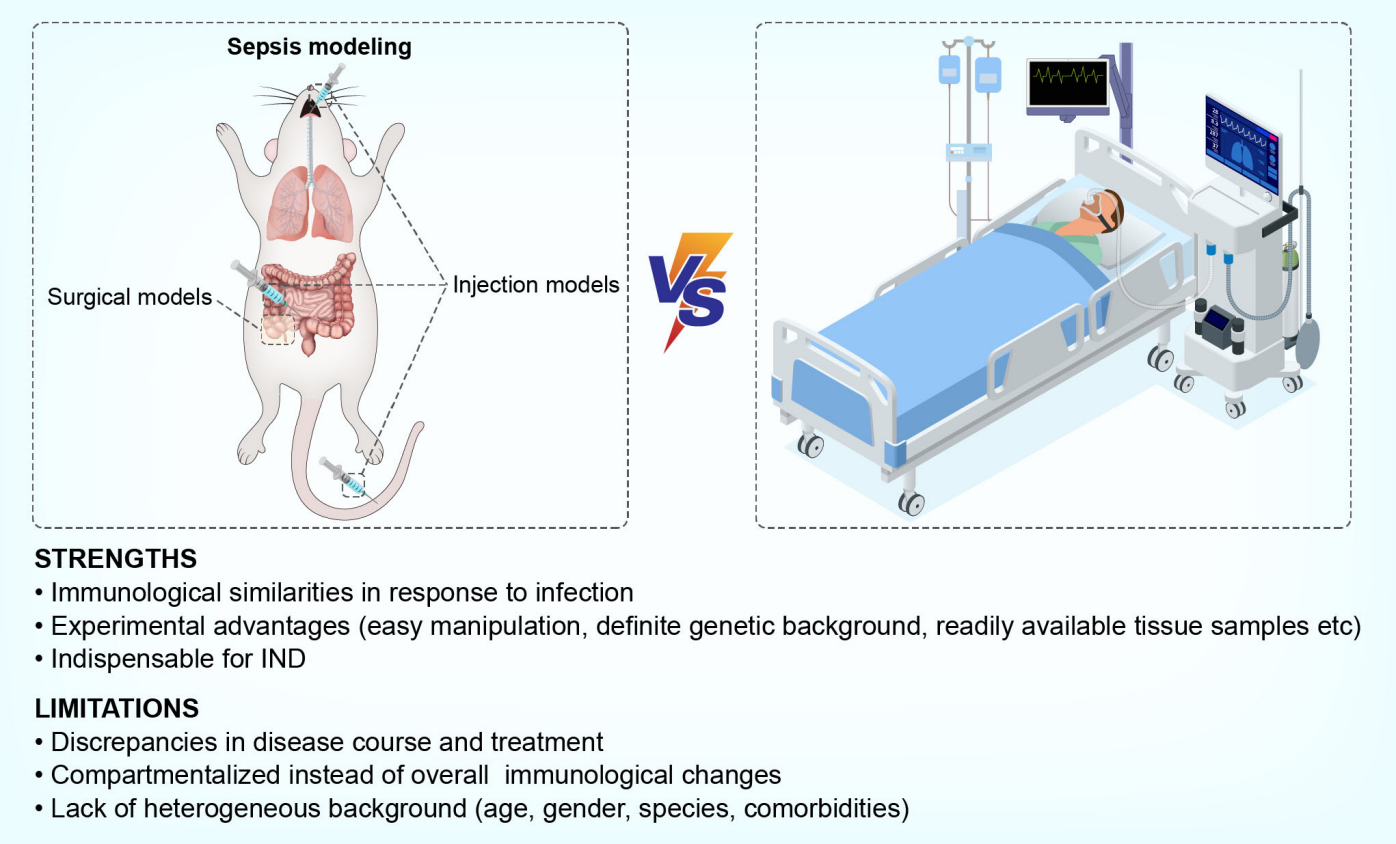
Strengths and limitations of using murine models to mimic sepsis-induced immunosuppression in humans.

Second, various methodological factors may dramatically alter the immune profiles of model mice. For example, it has been demonstrated that immunosuppression is well reproduced in the CLP model (11, 18) whereas other polymicrobial peritonitis models, such as the CASP model and the cecal slurry model, have ongoing proinflammatory responses that do not reach an immunosuppressive state (18, 76). Moreover, murine models are established in young, healthy, homogeneous, and pathogen-free animals, compared to the heterogeneous background conditions of human sepsis. Therefore, the common establishment of sepsis in these animals cannot fully replicate those of human sepsis, which occurs predominantly in a heterogeneous aging population, with existing comorbidities. Furthermore, individuals with sepsis harbor commensal microbiota or acquired infections that may dramatically affect their immunological state. These differences make it hard to replicate the exact features of immunosuppression in human sepsis by using standardized murine models. With regards to the comparison of murine models to human sepsis, another considerable discrepancy exists in post-sepsis interventions. Human patients with sepsis always receive supportive care, such as surgical resection, antibiotics, analgesia, and organ support. However, these interventions are absent or inconsistently performed during the care of human patients or in mouse models. Taking the use of analgesics as an example, opioids like buprenorphine, hydromorphone, oxycodone, and tramadol are less immunosuppressive than morphine and fentanyl, which suggests that different choices of analgesics may cause variations in the immune status of animals thereby affecting their immunological phenotypes during the modeling of sepsis (77). Finally, the design and execution of animal experiments lack inter-lab consistency, with sufficiently powered, randomized, or blinded analyses being rarely conducted (74). Therefore, positive outcomes are more likely to be reported, leading to a biased interpretation that limits the success of translation into human subjects (78). A brief description of strengths and limitations is depicted in **Figure 2**.

## Future directions to narrow the gaps between murine models and human sepsis

### Reducing experimental inconsistency

Standardized sepsis models are necessary to minimize discordant results between models and reduce discrepancies between animal and human sepsis (79). The recently published minimum quality threshold in pre-clinical sepsis studies (MQTiPSS), which was published by a consortium of experts from various research institutions, proposed a set of guidelines to enhance the consistency and translational value of sepsis models *via* the development of standardized technical procedures (80). In addition, we and other researchers have reported attempts to improve the consistency of CLP or CASP models by standardizing surgical procedures and the application of specialized surgical tools (81, 82). Nevertheless, we are some distance from creating standardized animal models that mimic the immunosuppression of human sepsis. Further research is thus needed to comprehensively document the immunological profiles of different septic models, such as the factors that predispose to immunosuppression development, the kinetics of immunological events, organ-specific immunological alterations and their association with organ dysfunction (7, 73). For example, a scoring system could be devised for predicting or evaluating the immunological parameters in sepsis, serving to reduce the inconsistencies in modeling and enable better comparisons between different experiments or laboratories (79).

### Increasing clinical relevance

Sepsis is highly heterogeneous in terms of pathogen species, genetics, age, gender, and comorbidities. It is therefore challenging to predict the progression from sepsis onset to immunosuppression. The complexity of sepsis calls for the development of a precision medicine approach, which requires more individualized and clinically compatible models for the recapitulation of sepsis-associated immunosuppression. For instance, the pathogen species used in current murine sepsis models are neither polymicrobial in nature nor clinically relevant. Therefore, a possible way of refining these models is to instead perform polymicrobial inoculation with clinical strains of microbes (83), or otherwise rebuild the microbiota of lab mice by introducing environmental pathogens prior to sepsis modeling (42). Additionally, most existing studies use young and healthy animals to model sepsis. Recently, attempts have been made to model sepsis in elderly mice, demonstrating remarkable differences in the immune response and disease outcomes when compared with their young counterparts (84, 85). In addition to considering the influence of age on sepsis pathology, future studies should aim to model sepsis in animals of different gender, or having a pre-existing illness (e.g., obesity and diabetes).

Sepsis often induces prolonged immunosuppression, which is associated with an increased risk of chronic dysfunction, such as weakness, secondary infection, and cancer (86). However, animals used to model septic often die too early for the onset of long-term consequences to be observed. The development of intensive care unit (ICU)-like murine facilities has already been shown prolong the survival of mice with sepsis and allow for longitudinal studies (87, 88).

Finally, translational research sepsis research to narrow the gaps when addressing therapeutic interventions for sepsis. Two recent systematic reviews have outlined recommendations for the design of preclinical studies to ensure clinical relevance (74, 89). Thus, in addition to the refinement of existing models, other experimental factors such as appropriate cohort selection, randomization, blinding, the timing of the intervention, as well as the use of powerful statistical analyses (i.e., using appropriate power and sample size), should be considered when designing clinically-relevant models of sepsis.

### Improving immunological similarities between mice and humans

In addition to the approaches outlined in earlier sections of this mini review, research is being carried out into improving the immunological similarities between mice and humans, thus narrowing the gap between these species for the study of sepsis. Sepsis is increasingly recognized as a syndrome of immunoparalysis rather than a cytokine storm-driven condition. A recent study reported that the switch from uncontrolled inflammation to ordered hypoinflammation and immunosuppression could be achieved by priming with a diverse pool of antigens to induce the activation of immunological memory (90). In this model, short-term mortality was reduced allowing for the long-term investigation of sepsis survivors, which may better resemble the course of sepsis-associated immunosuppression observed in humans (90). Environmental factors such as the microbiota also greatly affect the immune status of laboratory mice. To address this issue, a recent study created wildling mice by transferring C57BL/6 embryos into wild mice. These wildling mice demonstrated phenocopied human immune responses, and are therefore more suitable as candidate animals for investigating immunosuppression in sepsis (91).

Although sepsis modeling is predominantly performed in mice, translational gaps exist between mice and humans due to differences in their immune systems (92). To solve this issue, mice with a reconstituted human immune system or bearing active human immunogens have been created. These ‘humanized mice’ have been successfully utilized to study viral infection and transplantation and are expected to bridge the translational gap between mice and humans. Some recent studies have demonstrated that features of immunosuppression in sepsis, including bone marrow suppression (93), reduced cell surface marker expression (94), and increased apoptosis (95), are better replicated in humanized mice after CLP modeling. Although limitations exist and humanized mice cannot fully replicate human immunology in sepsis, this model may offer an alternative approach for the study of immunosuppression in sepsis. Given that the microbiome shapes human immunity and affects the outcomes of clinical sepsis, another approach would be to recreate a humanized microbiome in model mice, thereby increasing the utility of mice as a model organism by populating them with the same pathogens that are present in the human microbiome during sepsis (96).

## Conclusions

Mice remain the organism of choice for modelling sepsis. However, questions and doubts continue to be raised regarding the reliability of murine models in sepsis research, due to conflicting reports and negative translational outcomes. Sepsis is a heterogeneous syndrome, rendered even more complex by the period of immunosuppression that follows early-stage disease. Given that an ideal modeling strategy has not yet been developed, efforts should focus on refining current murine models to reduce inconsistency and increase clinical relevance; these improved murine models will be invaluable tools for study of the complex immunopathology of human sepsis.

## Author contributions

NW and XL reviewed literatures and wrote the manuscript. NW prepared figures and tables. YL reviewed literature and help to prepare figures. JZ and XL conceived the review article and made substantial revision before submission. All authors contributed to the article and approved the submission.

## Funding

This work was supported by grants from the National Natural Science Foundation (81873955, 81772137).

## Conflict of interest

The authors declare that the research was conducted in the absence of any commercial or financial relationships that could be construed as a potential conflict of interest.

## Publisher’s note

All claims expressed in this article are solely those of the authors and do not necessarily represent those of their affiliated organizations, or those of the publisher, the editors and the reviewers. Any product that may be evaluated in this article, or claim that may be made by its manufacturer, is not guaranteed or endorsed by the publisher.
